# Supplementary material for: Molecular understanding of the critical role of alkali metal cations in initiating CO2 electroreduction on Cu(100) surface
Source: Nat Commun. 2024 Jan 19;15:612. doi: 10.1038/s41467-024-44896-x (PMC10799043; doi:10.1038/s41467-024-44896-x)
Supplement: Supplementary file 1 — Supplementary Information [file 41467_2024_44896_MOESM1_ESM.pdf]

Supporting Information

Molecular Understanding of the Critical Role of Alkali Metal Cations in Initiating CO_2 Electroreduction on Cu(100) Surface

Zhichao Zhang[1], Hengyu Li[1], Yangfan Shao[1], Lin Gan[1], Feiyu Kang[1,*], Wenhui Duan[2,3,4], Heine Anton Hansen,[5] Jia Li[1,*]

[1]*Shenzhen Geim Graphene Center and Institute of Materials Research, Tsinghua Shenzhen International Graduate School, Tsinghua University, Shenzhen, 518055, People's Republic of China*

[2]*State Key Laboratory of Low Dimensional Quantum Physics and Department of Physics, Tsinghua University, Beijing 100084, People's Republic of China*

[3]*Institute for Advanced Study, Tsinghua University, Beijing 100084, People's Republic of China*

[4]*Frontier Science Center for Quantum Information, Beijing 100084, People's Republic of China*

[5]*Department of Energy Conversion and Storage, Technical University of Denmark, Kgs. Lyngby 2800, Denmark*

[*]Correspondence and request for materials should be sent to li.jia@sz.tsinghua.edu.cn, fykang@sz.tsinghua.edu.cn

Contents

1. Supplementary Figures 1
2. Supplementary Tables 5
3. Supplementary Notes 6
Supplementary Note 1. Model and potential control: use of a Ne counter electrode 6
Supplementary Note 2. Calculation of free energy profiles 8
Supplementary Note 3. Error estimation 10
Supplementary Note 4. Validation of stable configurations 11
Supplementary Note 5. Hydrogen bond lifetime 13
Supplementary Note 6. Constant potential correction for free energy barrier 14
Supplementary Note 7. Grand canonical simulations in implicit solvation 16
4. Supplementary References 17

1. Supplementary Figures

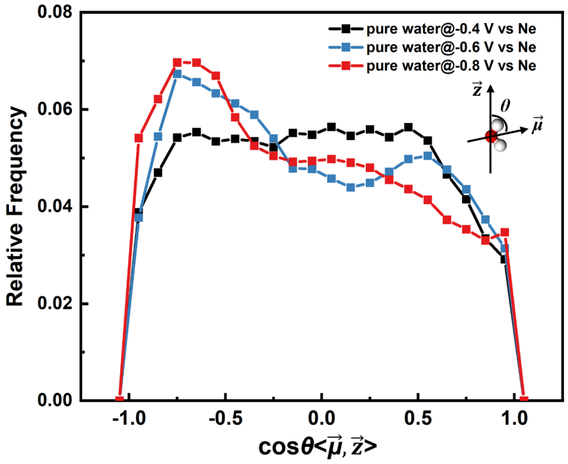

Supplementary Fig. 1 | Orientation distribution of water molecules under various potentials. Dipole distribution of water molecules at the Cu-water interface within 5 Å of the uppermost Cu layer under different applied potentials. $\vec{z}$ is the direction perpendicular to the interface, and $\vec{\mu}$ refers to the orientation of the water molecule. Along with the potential decrease, the water molecules are oriented to rotate the O-H bond toward the interface, shown as the transformation from a flat distribution to the one with a single peak.

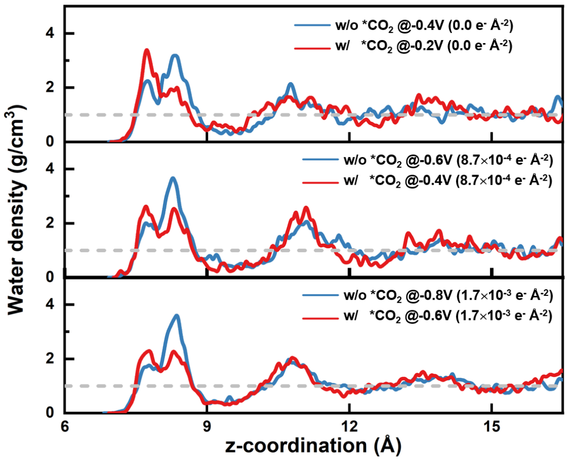

Supplementary Fig. 2 | Water density distributions under various potentials. Water density distribution along the *z*-axis perpendicular to interface for different applied potentials (represented by additional charge) and with/without a *CO_2 molecule at the Cu-water interface. The dashed gray lines indicate water density of approximately 1 g/cm^3, while blue and red lines correspond to distributions with and without *CO_2, respectively. The applied potentials are obtained from the workfunction of the whole system, referring to the neon counter electrode. (**Supplementary Note 1**).

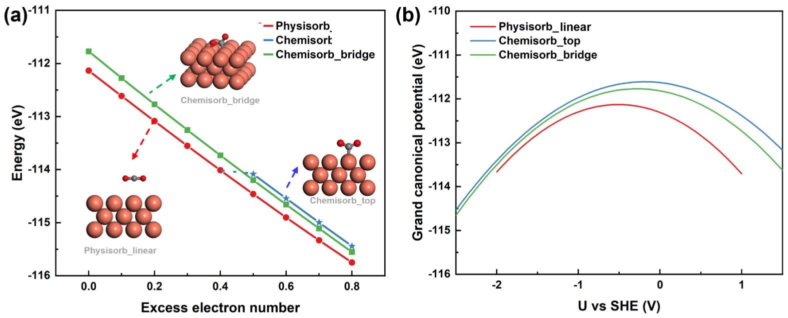

Supplementary Fig. 3 | Stable adsorption configurations of CO_2@Cu(100) in fully implicit solvent. (a) Under constant charge condition, the comparison of total energies between three representative adsorption configurations shown as illustrations, where blue, red and brown spheres represent Cu, O and C atoms, respectively. (b) Under constant potential condition, the comparison of grand canonical potentials between these three systems. (Methods of grand canonical calculations are shown in **Supplementary Note 7**)

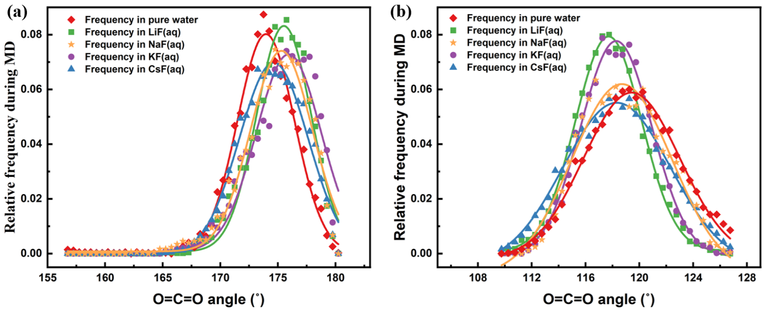

Supplementary Fig. 4 | Angle distributions of CO_2 molecule at initial states and final states in different solutions. Angle distribution of (a) a free CO_2 and (b) a chemisorbed $^*CO_2$ at the Cu-electrolyte interface with and without alkali metal cations. The solid lines represent the fitted curves to guide the viewer.

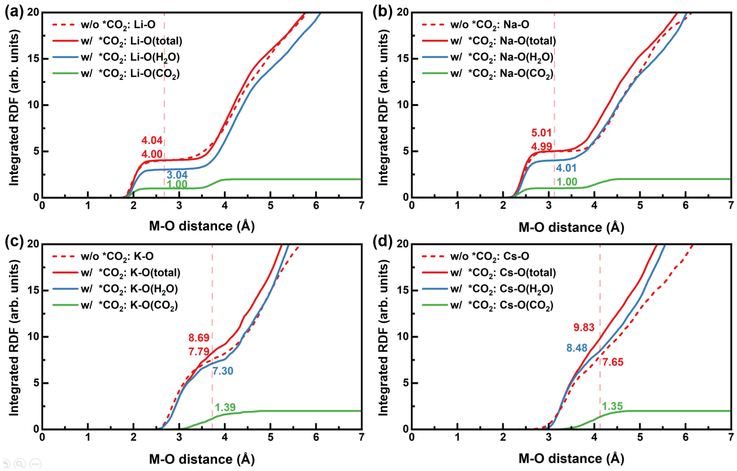

Supplementary Fig. 5 | Coordination analysis of the first solvation shell of M^+ at transition states. Integrated radial distribution function (RDF) for (a) Li-O, (b) Na-O, (c) K-O, and (d) Cs-O pairs at the Cu-electrolyte interface. The vertical pink dashed lines represent the radius of the first solvation shell of the respective cation. The numbers represent the total coordination

number of cations at the interface with and without the $*CO_2$. The coordination number of the cations at the interface with $*CO_2$ derived from H_2O and $*CO_2$ molecules are also shown for comparison.

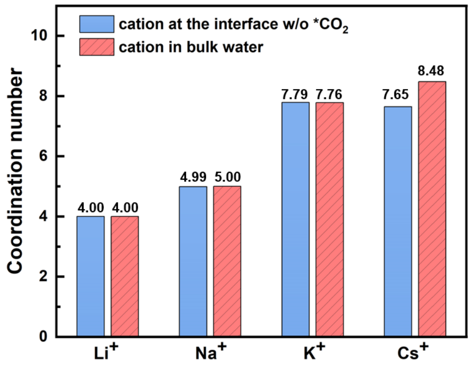

Supplementary Fig. 6 | Coordination number of M^+ in various environments. Integrated RDF of M^+-O pairs in bulk water (red) and near the Cu-electrolyte interface without $*CO_2$ (blue). Only Cs^+ would be partially desolvated at the interface.

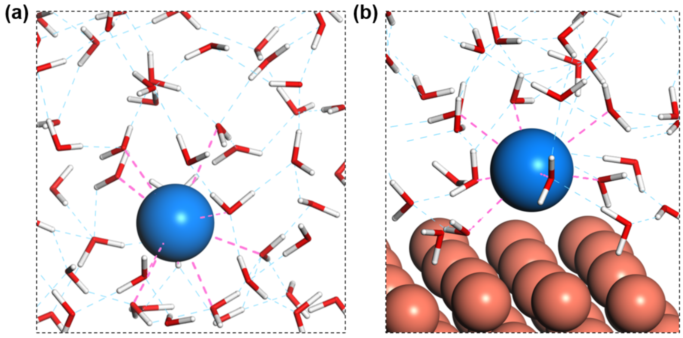

Supplementary Fig. 7 | Fully solvation and partial desolvation configurations of Cs^+. Representative snapshots of the solvation structures of Cs^+ (a) in bulk water and (b) near the Cu-electrolyte interface. The dashed red lines connect the Cs^+ ion and the water molecules in its first solvation shell.

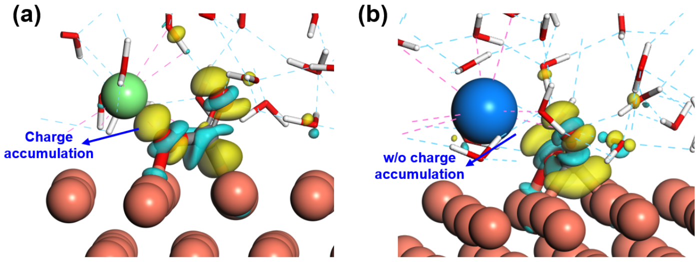

Supplementary Fig. 8 | Charge difference analysis between Li^+/Cs^+ and $*CO_2$. Charge differences between (a) Li^+ and $*CO_2$ and (b) Cs^+ and $*CO_2$. The yellow regions indicate regions of charge accumulation, and the light blue regions represents regions of charge depletion.

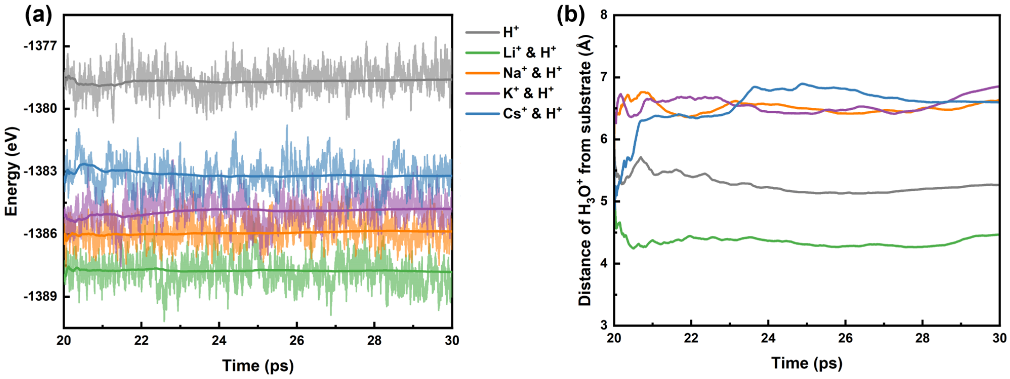

Supplementary Fig. 9 | Convergence of the systems with proton and various alkali metal cations. Cumulative average of total energy (a) and proton-interface distances (b) of the systems with various alkali metal cations during the sampling period. The equilibration criterion used in our study involves monitoring the energy drift of the systems less than 0.03 eV/ps and the motion of protons smaller than 0.1 Å/ps for a duration greater than 5 ps to ensure the convergence. The original results of energies are shown as lighter fluctuating lines in (a), and the original results of (b) correspond to Fig.5a in the main text. Detailed analysis is shown in Section 2.3 of the main text.

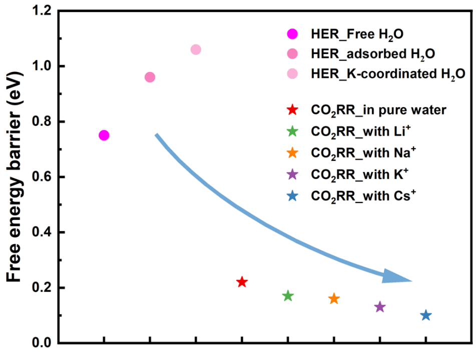

Supplementary Fig. 10 | Comparison of free energy barriers between HER and CO_2RR under various conditions. Free energy barriers of hydrogen evolution reaction (HER) with different proton sources at the Cu surface. Results of CO_2RR are also shown for comparison.

2. Supplementary Tables

Supplementary Table 1. CO_2 Adsorption enthalpy in pure water systems under various potentials

**U vs Ne electrode**	**ΔH_{ad} (eV)**
-0.6 V	-0.28
-0.8 V	-0.29
-1.0 V	-0.31

Supplementary Table 2. Typical and maximal coordination radius of the first solvation shell of M^+

**Bond "length"**	**1st Peak(Å)**	**1st valley(Å)**
Li-O	1.95	2.65
Na-O	2.45	3.15
K-O	2.90	3.75
Cs-O	3.25	4.15

Values are extracted from the same original dataset of Supplementary Fig. 5.

Supplementary Table 3. Adsorption free energy of CO_2 with different ions at PZC

**Ions**	**ΔG_{ad} (eV)**
Li^+	-0.86
Na^+	-0.69
K^+	-0.65
Cs^+	-0.69

PZC: potential of zero charge, $\approx$0.6 V_{SHE} in this case.

Supplementary Table 4. Charges that CO_2 carries at transition states with different cations

**Cations**	**Charger of CO_2 @TS**
Pure water	-0.34 *e*
Li^+	-0.25 *e*
Na^+	-0.24 *e*
K^+	-0.20 *e*
Cs^+	-0.17 *e*

Values are calculated with Bader charge analysis

3. Supplementary Notes

Supplementary Note 1. Model and potential control: use of a Ne counter electrode

Supplementary Fig. 11 shows the representative atomic model. To represent the electrode, we chose the Cu(100) surface modeled as a three-layer slab with the p(3×3) supercell. The space above the Cu surface was filled with 81 water molecules with a density of 1 g cm^{-3}. In order to maintain the water density, one water molecule was replaced with a CO_2 molecule. The volume of the solvent space was expanded with the addition of ions. Alkali metal cations M^+ (Li^+, Na^+, K^+, or Cs^+) were initially positioned ~5 Å away from the Cu(100) surface, while an anion F^- was placed far away on the other side of the solvent to form an ionization pair with the cation, in order to keep the applied potential close to the CO_2RR onset potential. An additional Ne atomic layer and a 12 Å vacuum layer were added over the interface model to act as a counter electrode and monitor the electrochemical potential of the entire system, as proposed by S. Surendralal *et al.* [1,2]

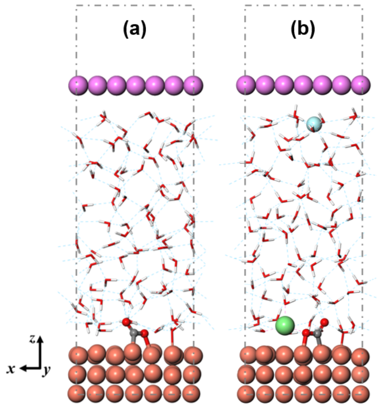

Supplementary Fig. 11 | Representative interfacial models. (a) Representative atomic model of Cu(100) surface with a $*CO_2$ covered with the water molecules and a counter a Ne counter electrode. (b) Representative atomic model with the addition of Li^+ and F^- ions. The green and light-blue balls represent the Li^+ and F^- ions, respectively.

The potential monitor method replies on the principles of density functional theory (DFT), which enforce an identical Fermi level throughout the system. By introducing a dipole correction along the *z*-axis in the vacuum layer,[3] the dipole moment (μ) of the entire system can be continuously monitored. This dipole moment is correlated with the work function difference between the two sides of the vacuum layer ($\Phi_{\text{Interface}}$ and Φ_{metal} in Supplementary Fig. 12), as represented by the following equation,

$$\Delta V = \frac{Q}{C} = \frac{4\pi\mu}{A} * k \qquad (1)$$

$$\Delta V = \Phi_{\text{Interface}} - \Phi_{\text{metal}} \qquad (2)$$

where μ is dipole moment of the system along the *z*-axis perpendicular to the interface, A is the surface area, and k is Coulomb constant (14.4 eV Å e^{-2}). In the Equation 2, Φ refers to work function of Ne (or the interface) or Cu side, respectively (Supplementary Fig. 12). Therefore, the applied potential can be determined once the work function of the metal side Φ_{metal} is known.

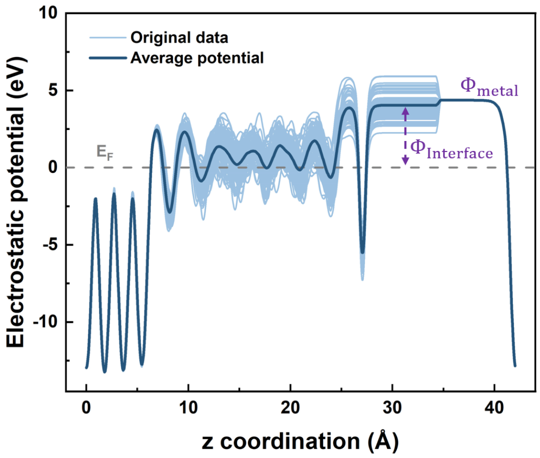

Supplementary Fig. 12 | Example of potential monitor method with workfunction calculations. Average electrostatic potential in the *xy* plane of a representative system as shown in Supplementary Fig. 11a. The light blue lines represent the original data from static calculations of a series of molecular dynamics snapshots, while the dark line represents the average value.

The work function of the metal slab (Φ_{metal}) can be considered constant, as shown in Supplementary Fig. 12. This assumption is reasonable due to the electrostatic screening ability of metal slabs. Consequently, $\Phi_{\text{Interface}}$ could be continuously monitored on-the-fly and transformed into applied potential (U) using Equation 3,

$$U \text{ vs SHE} = \Phi_{\text{Interface}} - 4.44 \quad (3)$$

The counter electrode model can also be utilized to charge the interfaces by adding or removing electrons on the Ne electrode, as shown in Fig.1 of the main text. This approach is based on the principle that DFT calculations enforce an identical Fermi level throughout the system as well. Thus, adding or removing electrons from Ne counter electrode results in additional or missing electrons in the Cu electrode, approximating a grand canonical ensemble of electrons. However, it is important to note that in these situations, the applied potential cannot be transformed to the SHE scale using Equation 3, as ΔV would take contributions from two charged interfaces (Ne-water and Cu-water) into account. Therefore, we chose another physical quantity of U_{Ne} (potential difference with Ne electrode), equal to ΔV, instead of U_{SHE} (potential versus SHE) to represent the applied potential in Fig. 1.

Supplementary Note 2. Calculation of free energy profiles

While the enthalpy is a common measure of a chemical reaction, reaction free energy is a more accurate descriptor of its feasibility, although it cannot be obtained directly from a standard molecular dynamic (MD) simulation. In this study, we used thermodynamic integration methods (Equation 4) to produce free energy profiles (see Fig. 2 and Fig. 3 in the main text) with the angle of CO_2 as the chosen collective variable (CV, denoted by ξ).

$$\Delta A_{\xi_1 \to \xi_2} = \int_{\xi_1}^{\xi_2} \mathrm{d}\xi^* (\frac{\partial A}{\partial \xi})_{\xi^*} \qquad (4)$$

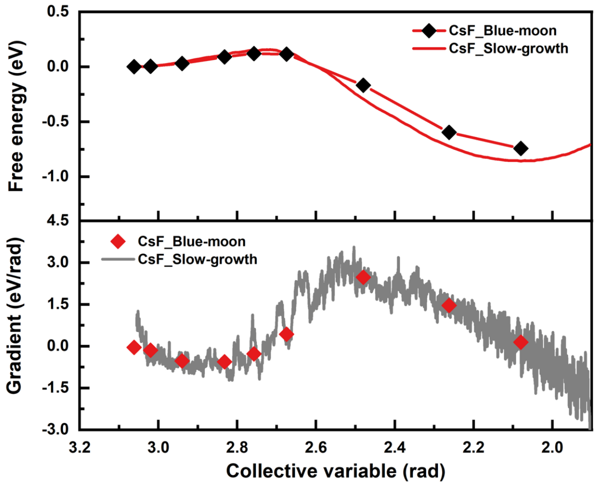

Supplementary Fig. 13 | Comparison of free energy profiles using various methods. Free energy profiles calculated during the CO_2 adsorption process with the addition of alkali metal cation Cs^+ calculated using both the slow-growth method and the blue-moon method.

To obtain rough positions of the initial states (IS), transition states (TS) and final states (FS), we employed the slow-growth method[4] with a constant transformation velocity of CV ($\dot{\xi} = 1 \times 10^{-4}$). However, due to the limited number of samples at each CV values in the slow-growth method (as shown in Supplementary Fig. 13), we utilized the blue-moon ensemble sampling[5] to generate more accurate results. Additionally, standard errors can be estimated for each point in the blue-moon method.

To obtain the free energy gradients, constrained molecular dynamic (cMD) calculations were performed at a series of selected CV previously determined from slow-growth calculations, using SHAKE algorithm[6]. The resulting free energy gradient of the constrained system was converted to that of the corresponding unbiased system using the blue-moon correction, as shown in the following (Equation 5),

$$\nabla A(\xi)|_{\xi=\xi^*} = \frac{\langle |\mathbf{Z}|^{-1/2} \cdot \nabla A(\xi^*) \rangle_{\xi^*}}{\langle |\mathbf{Z}|^{-1/2} \rangle_{\xi^*}} \qquad (5)$$

where $\nabla A(\xi)$ represents the free energy gradient in an unbiased system, and ξ^* represent the constrained system with the reaction coordinate ξ^*, i.e., $\dot{\xi} = 0$ in corresponding cMD simulation. The angular parentheses represent the statistical average of the quantity enclosed in them. $\mathbf{Z} = \sum_{i=1}^{M} \frac{1}{m_i} \left(\frac{\partial \xi}{\partial x_i}\right)^2$ represents the mass metric tensor in the system with M degrees of freedom.

Each free energy gradient generated from the cMD simulation is averaged after the cumulative average of gradient reaches a steady state for at least 3 ps. Note that in the K^+-*CO_2 complex systems,

K^+ fluctuates between the side and bridge configuration with $*CO_2$. In this case, contributions from both configurations are considered (detailed discussion is shown in Supplementary Note 4). The free energy profile is then obtained by integration according to Equation 4. An example of this procedure is shown in Supplementary Fig. 13.

In this study, we use a one-dimensional CV: $\xi = \text{angle of } CO_2$. The reasons are as follows: We have considered two possible collective variables. One is the possibly more commonly used bond length of Cu-C bond (C is from CO_2), and the other is the CO_2 angle. But as we performed MD simulations at initial states (flat and physisorbed CO_2) and final states (bent and chemisorbed $*CO_2$) with various cations to discover the key contributions to the differences of initial/final states, we found that the CO_2 angle is relatively more stable than Cu-C bond length on Cu(100) surface, and thus could be more suitable to describe the reaction coordinates, i.e., to be the collective variable. For initial states with a physisorbed CO_2, the CO_2 molecule would rotate or translate at ~3.4 Å away from the interface along with water dynamics, so we cannot simply determine which Cu atom it would tend to adsorb on due to the weak attraction by vdW interaction. However, the CO_2 angle would always fluctuate ~175°±3° with a Gaussian distribution (Supplementary Figure 4). This is a little different from the case of single atom catalysts (SAC). In SAC systems, the adsorption site is pre-determined, which allows us to find a simple constraint (like C-Metal bond) to be the collective variable. Furthermore, the adsorption configuration of $*CO_2$ at final states further convinced us to choose CO_2 angle as the collective variable. The bent $*CO_2$ adsorbs at the Cu(100) surface with both C-Cu and O-Cu bonds, and the C atom is adsorbed at bridge site instead of atop site like we traditionally considered in implicit solvation. This means a single C-Cu bond cannot fully describe the achievement of final state. However, the CO_2 angle would still obey a Gaussian distribution centered at ~119°±3°, which provide a good description of the reaction coordinate.

Supplementary Note 3. Error estimation

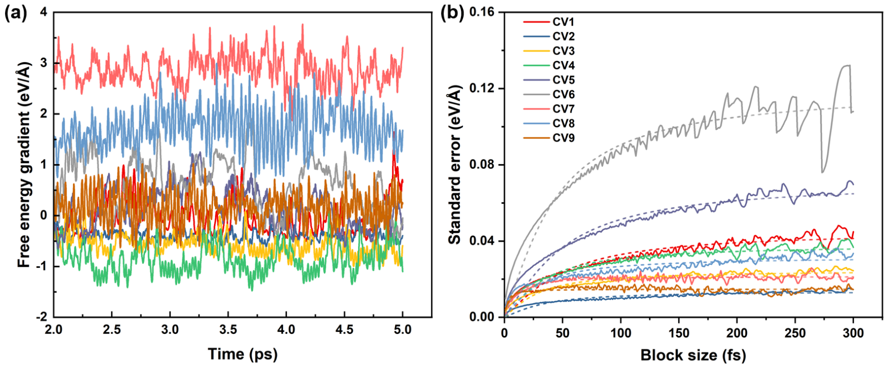

Supplementary Fig. 14 | Block average and error estimation. (a) Original data of the free energy gradient results corresponding to nine different collective variables (CVs) along CO_2 bending process with the addition of Li^+ cation. (b) Error estimation using the block average method. The solid lines are block average results from (a), while the dashed lines represent the fitted results with arctangent functions.

Traditional error estimation methods are not suitable for time-correlated data, such as the free energy gradients obtained using the cMD and the blue-moon method in this study. Therefore, we used the block average method to estimate the errors. This method involves gradually increasing the f block size, and the standard errors of a group of correlated data would level off as the block size increases. In our study, we fit the results with the arctangent functions and used the asymptote as corresponding maximum error value. An example of the original data and the fitted function is shown in Supplementary Fig. 14a and Supplementary Fig. 14b, respectively. After obtaining the standard error of the free energy gradient at each CV, we calculated the errors for free energy profiles through integration. These errors are represented by the shaded regions in Fig. 3 of the main text.

Supplementary Note 4. Validation of stable configurations

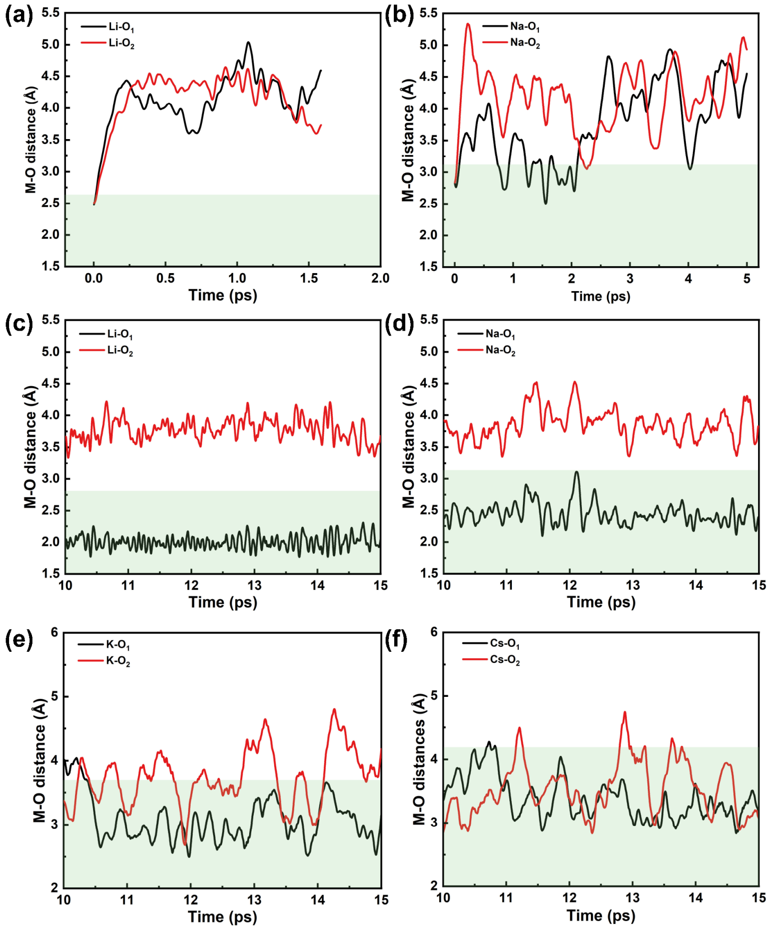

Supplementary Fig. 15 | Confirmation of stable configurations of M^+-*CO_2 complex. (a-b) Initializing from bridge configuration, the distances between (a) Li^+, (b) Na^+ and O atoms of *CO_2. (c-d) Initializing from side configuration, the distances between (c) Li^+, (d) Na^+ and O atoms of *CO_2. (e-f) Initializing from bridge configurations, the distances between (e) K^+, (f) Cs^+ and O atoms of *CO_2.

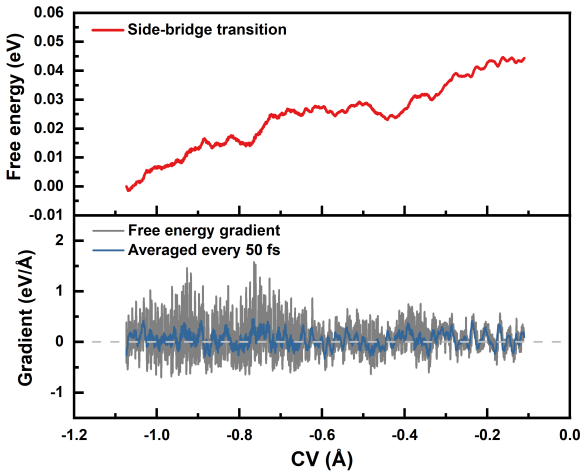

Supplementary Fig. 16 | Transition between side and bridge configurations of K^+-*CO_2 complex. Free energy profile during the process that K-*CO_2 complex move from side to bridge configuration. The barrier of as low as ~0.05 eV.

To avoid trapping the Li^+ or Na^+ cation in a local minimum around the side configuration, which could lead to uncertainty in the initial structure choice, we also tried to initiate the reaction pathway from the bridge configuration. The Li^+ or Na^+ cations quickly move away from the bridge configuration, simultaneously forming a new solvation shell, and fail to reach a stable configuration that coordinates with both O atoms of $*CO_2$ (Supplementary Fig. 15a-b). In contrast, when initialized from the side configuration, Li^+ and Na^+ could coordinate very stably with one of the O atoms from $*CO_2$ (Supplementary Fig. 15c-d). Therefore, we could conclude that Li^+ and Na^+ cations prefer to coordinate with $*CO_2$ by side configuration.

In comparison, larger cations show relatively stable coordination with $*CO_2$ through the bridge configuration (Supplementary Fig. 15e-f). In particular, Cs^+ coordinate with $*CO_2$ through the bridge configuration more stably, while K^+ would move between side and bridge configurations during the simulation, which could also be observed as the broad distribution of K^+-O distances in Fig. 4b of the main text. To confirm this phenomenon, we calculated the free energy barrier of the transition between side and bridge configurations of the K^+-$*CO_2$ complex. We found that this barrier is pretty small (~0.05 eV, Supplementary Fig. 16). Therefore, we included both the contributions from side and bridge configurations in the free energy calculations for the K^+ cation.

Supplementary Note 5. Hydrogen bond lifetime

To evaluate the lifetime of hydrogen bond in systems containing different alkali metal cations and/or a proton, we selected the trajectories from the last 10 ps of each simulation as a representative sample. All calculated hydrogen bond lifetimes are shown in Supplementary Table 5, where detailed methods described below the table.

Supplementary Table 5. Hydrogen bond lifetime with different cations

System	Continuous H-bond lifetime $\tau_C(ps)$	Intermittent H-bond lifetime $\tau_I(ps)$
H^+	0.19	3.62
H^+&Li^+	0.16	2.44
H^+&Na^+	0.16	2.17
H^+&K^+	0.13	1.45
H^+&Cs^+	0.12	1.05

Note: The hydrogen bond lifetime is estimated using the time autocorrelation function,[7,8]

$$C(t) = \langle \frac{\sum h_{ij}(t_0)h_{ij}(t_0+\tau)}{\sum h_{ij}(t_0)^2} \rangle \quad (6)$$

Here, h_{ij} indicates the presence of a hydrogen bond between atoms ij: if a hydrogen bond exists, $h_{ij} = 1$; otherwise, $h_{ij} = 0$. τ is the time variable to determine whether a specific hydrogen bond still exits. t_0 is the time origin, and the average (indicated by angular bracket) is taken over a series of time origins with an interval of Δt, where Δt is chosen to be 1 ps. The results of the autocorrelation functions were fitted with the biexponential function,

$$C(t) = Aexp(-t/\tau_1) + Bexp(-t/\tau_2) \quad (7)$$

The average hydrogen bond lifetime is determined as $A\tau_1 + B\tau_2$. In addition, intermittent hydrogen bond lifetimes are also calculated with a tolerant time of 0.2 ps. This allows a hydrogen bond to be considered present even if it breaks up to the specified time scale.

Supplementary Note 6. Constant potential correction for free energy barrier

There is a common issue in the simulation of electrochemical reactions that needs to be discussed about constant potential and constant charge conditions. In traditional DFT calculations, systems are kept charge-neutral, with the total number of charges remaining unchanged. In experiments, however, the applied potential is maintained, which means that the number of charges can fluctuate and be exchanged with an external electron reservoir, i.e., the potentiostat. For instance, as schematically shown in Fig. 1d of main text, in the charge-neutral state, the electron density redistribution and the dipole of the bent CO_2 induce a potential increase of ~0.2 V, resulting in a higher coverage of chemisorbed water molecules (Fig. 1b and Const-Q case in Fig. 1d of main text). However, when the number of charges on the Cu surface is dynamically adjusted to keep a constant potential, the intensity of the first peak is almost identical to the reference (Fig. 1c and Const-U case in Fig. 1d of main text). Note that the decrease of the second peak is due to the replacement of the water molecule by the chemisorbed $*CO_2$. This observation emphasizes the importance of potential control in electrochemical simulations. The potential change along the reaction path could affect the arrangement of the interfacial water, which could influence the subsequent proton transfer process of CO_2RR and competing HER, considering the proton donor possibly from chemisorbed or physisorbed water.

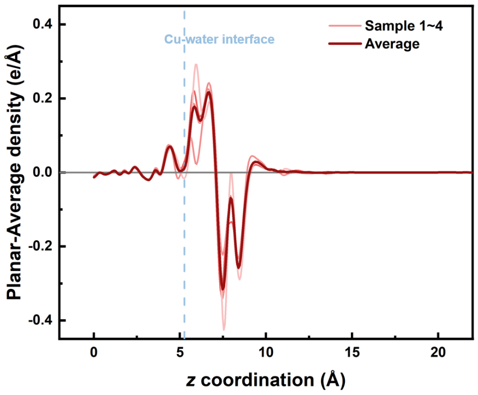

Supplementary Fig. 17 | Charge transfer at solid-liquid interface. Planar-averaged charge difference of a Cu-water interfacial system, showing the amount of charge transfer between the Cu substrate and the first water layer. Positive charge density indicates electron accumulation, while negative charge density indicates electron depletion.

In fact, the potential change during the CO_2 activation process in this work is not significant, only about ~0.2 V, compared to reactions involving bond breaking. This observation can be explained by considering the contributions to the interfacial potential drop at the EDL. When a metal electrode is in contact with water, charge transfer between the water layer and the substrate occurs, resulting in a large dipole and an electrified interface (Supplementary Fig. 17). The interfacial dipole dominates the total potential drop. For the system with a $*CO_2^-$ on the Cu surface, the transferred electron can still be considered as part of the substrate due to the Frumkin adsorption behavior of $*CO_2^-$ within 3 Å of the interface in the inner Helmholtz plane. Therefore, the potential change with a transferred electron is not significant. Nevertheless, we want to emphasize this issue due to its potential impact on the interfacial structures. This information is crucial for a comprehensive understanding of electro-chemical reactions.

Despite this, a constant-potential method for AIMD calculations has yet to gain widespread

acceptance, although it has almost become a common practice for static calculations with the implicit solvent.[10,11] In this work, we adopted a post-treatment correction using the surface charge method proposed by K. Chan and J. K. Nørskov.[9,12,13] By obtaining the surface charge q and corresponding potential Φ for IS and TS states, the free energy barrier under two different potentials could be converted to the one under an identical potential using the following equation,

$$E_{\mathrm{TS}}(\Phi_{\mathrm{IS}}) - E_{\mathrm{IS}}(\Phi_{\mathrm{IS}}) = E_{\mathrm{TS}}(\Phi_{\mathrm{TS}}) - E_{\mathrm{IS}}(\Phi_{\mathrm{IS}}) + \Delta E_{\mathrm{corr}} \quad (8)$$

$$\Delta E_{\mathrm{corr}} = \frac{1}{2}(q_{\mathrm{TS}} - q_{\mathrm{IS}})(\Phi_{\mathrm{TS}} - \Phi_{\mathrm{IS}}) \quad (9)$$

where q is estimated using Bader charge analysis, and Φ could be obtained using the potential monitor method introduced previously. The corrections for free energy barriers of the five systems are shown in Supplementary Table 6.

Supplementary Table 6. Constant potential corrections for free energy barriers of CO_2 adsorption

	q_{TS}	q_{IS}	Φ_{TS} (eV)	Φ_{IS} (eV)	**Barrier (eV)**	**Corrected barrier (eV)**
**pure**	593.747	593.523	3.754	4.082	**0.175**	**0.212**
**Li^+**	593.785	593.646	4.068	4.325	**0.153**	**0.171**
**Na^+**	593.768	593.639	3.779	3.999	**0.143**	**0.157**
**K^+**	593.714	593.654	3.546	3.808	**0.113**	**0.122**
**Cs^+**	593.764	593.669	4.188	3.601	**0.120**	**0.09**

Supplementary Note 7. Grand canonical simulations in implicit solvation

In order to further clarify the adsorption configuration difference in implicit and explicit solvation, we evaluated the possible adsorption configurations in implicit solvent under applied potential with the help of grand canonical calculations.[11,14]

Here we calculate the grand canonical potentials using the quadratic fitting method derived from constant-charge results. Firstly, a series of additional charges (q) are introduced into the simulation cell to adjust the fermi level as well as the applied potential, and then a fitted quadratic curve could be obtained between the free energies $F(q)$ and charge q.

$$F(q) = aq^2 + bq + c \quad (10)$$

where $q = n - n_0$, n is total electron number under a specific applied potential, and n_0 is the total electron number at neutral case, i.e., at PZC. The a, b, c are fitted parameters.

Next, grand canonical potential $G(q, U)$ could be obtained from the relation between $G(q, U)$ and $F(q)$:

$$G(q, U) = F(q) - q\mu \quad (11)$$

where $\mu = -eU + \mu_{SHE}$, μ is the chemical potential of electron, e is the unit charge of electron, U is the applied potential versus standard hydrogen electrode (SHE), and $\mu_{SHE} = -4.6$ eV is the reference chemical potential value at SHE.

According to the minimization process, $\frac{dG(q,U)}{dq} = 0 \Rightarrow q = -\frac{eU+b-\mu_{SHE}}{2a}$. Combining with Equation 10-11, we can get the final relation between grand canonical potential with applied potential,

$$GCP(U) = -\frac{1}{4a}(eU + b - \mu_{SHE})^2 + c \quad (12)$$

Herein, we considered three typical CO_2 adsorption configurations, i.e., physisorbed linear CO_2, chemisorbed *CO at atop site with a single C-Cu bond formation and both O-atoms symmetrically facing upward, and the bidentate configuration that is the same as in explicit solvent. The results shows that the physisorbed linear CO_2 is indeed dominant at a wide potential range in implicit solvent, which has reached the CO_2RR active region (Supplementary Figure 3). Specifically, the bidentate configuration could energetically less stable than the physisorbed CO_2 by over 0.3 eV at the potential of -0.6 V_{SHE}, which is the concerned potential range in our manuscript. This difference obviously indicates the vital role of hydrogen bond of stabilizing $*CO_2$ at Cu(100), and thus raises the need to reevaluate previously proposed mechanisms, especially those involving interactions between intermediates and the surrounding electrochemical environment under implicit solvation conditions. While thermodynamic simulations can still provide approximate estimates, it is imperative to conduct a careful investigation of reaction mechanisms at the molecular level.

4. Supplementary References

1. Surendralal, S. *et al.* Impact of water coadsorption on the electrode potential of H-Pt(111)-liquid water interfaces. *Phys. Rev. Lett.* **126**, 166802 (2021).
2. Surendralal, S. *et al.* First-principles approach to model electrochemical reactions: Understanding the fundamental mechanisms behind Mg corrosion. *Phys. Rev. Lett.* **120**, 246801 (2018).
3. Neugebauer, J. & Scheffler, M. Adsorbate-substrate and adsorbate-adsorbate interactions of Na and K adlayers on Al(111). *Phys. Rev. B* **46**, 16067-16080 (1992).
4. Woo, T. K. *et al.* A combined Car−Parrinello QM/MM implementation for ab initio molecular dynamics simulations of extended systems: Application to transition metal catalysis. *J. Phys. Chem. B* **101**, 7877-7880 (1997).
5. Bucko, T. Ab initiocalculations of free-energy reaction barriers. *J. Phys.: Condens. Matter* **20**, 064211 (2008).
6. Ryckaert, J.-P. *et al.* Numerical integration of the cartesian equations of motion of a system with constraints: molecular dynamics of n-alkanes. *J. Comput. Phys.* **23**, 327-341 (1977).
7. Gowers, R. J. & Carbone, P. A multiscale approach to model hydrogen bonding: The case of polyamide. *J. Chem. Phys.* **142** (2015).
8. Smith, P. *et al.* On the interaction of hyaluronic acid with synovial fluid lipid membranes. *Phys. Chem. Chem. Phys.* **21**, 9845-9857 (2019).
9. Chan, K. & Nørskov, J. K. Electrochemical barriers made simple. *J. Phys. Chem. Lett.* **6**, 2663-2668 (2015).
10. Vijay, S. *et al.* Dipole-Field Interactions Determine the CO_2 Reduction Activity of 2D Fe–N–C Single-Atom Catalysts. *ACS Catal.* **10**, 7826-7835 (2020).
11. Hossain, M. D. *et al.* Reaction mechanism and kinetics for CO_2 reduction on nickel single atom catalysts from quantum mechanics. *Nat. Commun.* **11**, 2256 (2020).
12. Chan, K. & Nørskov, J. K. Potential dependence of electrochemical barriers from ab initio calculations. *J. Phys. Chem. Lett.* **7**, 1686-1690 (2016).
13. Gauthier, J. A. *et al.* Unified Approach to Implicit and Explicit Solvent Simulations of Electrochemical Reaction Energetics. *J. Chem. Theory Comput.* **15**, 6895-6906 (2019).
14. Huang, Y. *et al.* Reaction Mechanism for the Hydrogen Evolution Reaction on the Basal Plane Sulfur Vacancy Site of MoS_2 Using Grand Canonical Potential Kinetics. *J. Am. Chem. Soc.* **140**, 16773-16782 (2018).
